# Validation and assessment of the self-injurious behavior scale for tic disorders (SIBS-T)

**DOI:** 10.1038/s41598-024-66528-6

**Published:** 2024-07-31

**Authors:** Natalia Szejko, Heike große Schlarmann, Anna Pisarenko, Martina Haas, Valerie Brandt, Ewgeni Jakubovski, Kirsten R. Müller-Vahl

**Affiliations:** 1 Clinic of Psychiatry, Social Psychiatry and Psychotherapy, Hannover Medical School, Carl-Neuberg-Str. 1, 30625 Hannover, Germany; 2 Department of Clinical Neurosciences, University of Calgary, Calgary, AB Canada; 3 Department of Bioethics, Medical University of Warsaw, Warsaw, Poland; 4 Centre for Innovation in Mental Health, University of Southampton, Southampton, UK

**Keywords:** Medical research, Neurology

## Abstract

Self-injurious behavior (SIB) is a well-known phenomenon in patients with chronic tic disorders (CTD). To investigate prospectively symptomatology of SIB in adults with CTD, we developed and validated the self-injurious behavior scale for tic disorders (SIBS-T). Patients completed the SIBS-T and a variety of assessments for tics and comorbidities. We investigated SIB frequency, internal consistency of the SIBS-T, and carried out an exploratory factor analysis (EFA). We enrolled n = 123 adult patients with CTD. SIB was reported by n = 103 patients (83.7%). The most frequently reported SIB were beating/pushing/throwing and were found in 79.6% of cases. Patients with SIB had significantly higher tic severity measured with the Adult Tic Questionnaire (ATQ) (*p* = 0.002) as well as higher severity of psychiatric comorbidities such as obsessive–compulsive symptoms (OCS) (*p* < 0.001,), attention deficit/hyperactivity disorder (ADHD) (*p* < 0.001,), and anxiety (*p* = 0.001). In addition, patients with SIB had significantly lower quality of life (*p* = 0.002). Pearson correlations demonstrated significant associations between SIB and severity of tics (*p* < 0.001), depression (*p* = 0.005), ADHD (*p* = 0.008), and borderline personality traits (*p* = 0.014). Consequently, higher SIBS-T also correlated with greater impairment of quality of life (*p* < 0.001). The internal consistency of the SIBS-T was good (α = 0.88). The EFA confirmed a single factor underlying the SIBS-T.

## Introduction

Tourette syndrome (TS) is characterized by the presence of motor and vocal tics starting before the age of 18. In the majority of patients, psychiatric symptoms co-exist, most commonly obsessive–compulsive symptoms (OCS) and disorder (OCD) and attention deficit/hyperactivity disorder (ADHD)^1^. Among further comorbidities including depression and anxiety, self-injurious behavior (SIB) is a well-known co-occurring symptom that may cause relevant impairment in affected patients^2^. However, so far there is no widely accepted definition of SIB and, as a result, various studies reported different prevalence rates in TS populations ranging from 4% up to 44%^2–6^. Furthermore, there is no general consensus regarding the term that should be used to describe this phenomenon resulting in various terminologies including self-injurious behavior^3–7^, self-harm behavior^2^, self-mutilation^8–10^, and autoaggression^11^.

In the largest study investigating SIB in TS, Mathews et al.^3^ enrolled n = 297 patients and found an association between SIB and OCD and impaired impulse control. Just recently, a group from Houston^5^ published another large study with n = 201 patients with TS, 34 of whom (16.9%) had comorbid SIB. The majority of patients experienced self-inflicted damage (11.4%), while only 3.5% of participants also had inclination to aggression towards external objects (e.g. destruction of furniture) and only 2% showed “tic-related SIB”. Individuals with SIB were found to have more complex motor tics, more OCS, greater tic severity, and to receive surgical treatment with deep brain stimulation^5^. A group from Poland^2^ investigated the prevalence and clinical correlates of SIB in 165 children and adults with TS. All in all, prevalence of SIB was relatively high in this sample and comprised 39.4%. The authors found an association not only with comorbid OCD and tic severity, but also with ADHD. In most patients, SIB started in childhood or adolescence (92.3%) and only rarely in adulthood (7.7%). In a large single centre sample (n = 1,032, n = 529 children and n = 503 adults), Sambrani et al.^12^ found a similar prevalence rate of SIB of 39%. SIB was highly associated with complex motor tics and coprophenomena. Kano et al.^13^ investigated comorbid symptoms in n = 88 children and adults with TS and found higher rates of SIB in those patients with comorbid OCD and ADHD. In a similar study, Eapen et al.^14^ included 91 consecutive adult patients with TS and reported a prevalence rate of 43.9%. SIB was found to correlate with OCS. In a study including 90 adults and children with TS, Robertson et al.^15^ found a relationship between SIB and obsessive trait of hostility. Freeman et al.^16^ conducted an international study comprising 3,500 children and adults with TS in which SIB was found in 14%, but with a wide range among centres (4–43%). Remarkably, females (18%) were significantly more often affected than males (13%). Again, SIB was found more frequently in individuals with comorbid OCD but was also associated with the number of comorbidities. Chen et al.^17^ examined the National Health Insurance Research Database of Taiwan and compared the risk of traumatic brain injury in 2,261 adults and children with TS to 20,349 healthy controls. Patients with TS and comorbid ADHD, OCD, and depression had an increased risk of injuries, while the use of antipsychotics was found to be protective. The risk of brain injuries correlated with violent motor tics and severe SIB.

In 2007, Cheung et al. introduced the term “malignant TS”^18^. It was defined as a condition causing two or more emergency room visits or one or more hospitalizations due to tics or associated comorbidities. During a 3-year follow-up period, 17 of 333 patients (5.1%) met the criteria for “malignant TS”. Hospital admission or emergency room visits were required because of tic-related injuries, SIB, uncontrollable violence and temper, or suicidal ideation/attempts. Compared with patients with “non-malignant TS”, those with “malignant TS” were significantly more likely to have a history of OCS/OCD, complex vocal tics including coprolalia, copropraxia, mood disorder, suicidal ideation, and poor response to medications. “Malignant TS” correlated with greater severity of motor tics and the presence of two or more comorbidities. The authors concluded that in particular OCS/OCD may play a central role in “malignant TS”.

Finally, a limited number of case reports has been published describing single patients with TS with specific kinds or extremely severe SIB. Krüger and Müller-Vahl^7^ described a 12-year-old boy who self-extracted four of his teeth. Fasano and Galluccio^19^ presented a case of a 17-year-old male patient who suffered from severe head tics that led to head trauma with subdural and subarachnoid hemorrhage followed by massive edema managed with decompressive craniotomy. Hood et al.^20^ reported a case of a 16-year-old adolescent with self-inflicted oral lacerations. However, based on current literature, severe SIB is a rare presentation in TS occurring in only about 4% to 5% of patients^3,18^.

Although SIB is a relatively common comorbidity in TS, until today, no generally accepted definition is available, the prevalence rate is unclear, and no standardized and validated measurement is available for the diagnosis and the assessment of SIB severity. Recent studies are limited by small sample sizes, imprecise definitions of SIB, and/or insufficiently objective evaluations of SIB severity mainly based on the vague criterion of “physical injury”. For example, Mathews et al.^3^ distinguished only between “mild” and “severe” SIB, whereby “severe” was defined by permanent tissue damage. Similarly, Szejko et al.^2^ differentiated between “mild” (no influence on everyday functioning and not causing any permanent tissue damage), “moderate” (visible physical injuries, for example, bruises or wound, but without need of medical assistance), and “severe” (causing psychological or physical impairment and requiring some sort of medical attention) SIB based on clinical judgement.

The aim of the present study was to develop a standardized instrument to measure for the first time prospectively type and severity of SIB in a large group of adult patients with TS and other chronic tic disorders (CTD). In addition, we investigated the psychometric properties of the newly developed the self-injurious behavior scale for tic disorders (SIBS-T). Finally, we were interested in determining whether SIB should be regarded as a manifestation of OCS/OCD or complex motor tics, or an independent comorbid phenomenon.

## Results

### Participants

We enrolled n = 123 adult patients (n = 82 (66.7%) males), mean age = 36.5 (SD = 14.18); n = 102 (82.9%) had prediagnosed TS, n = 13 (10.6%) CTD, and n = 8 (6.8%) unspecified tic disorder. Nearly half of the patients (n = 55, 44.7%) indicated having been diagnosed in the Tourette Outpatient Clinic at MHH. More than half of patients (56.1%, n = 69) were taking medications for tics and/or psychiatric comorbidities, most frequently aripiprazole (n = 20, 29%) and cannabis-based medicines (n = 17, 24.6%). The most frequently self-reported psychiatric diagnoses were depression (n = 33, 26.8%), OCD (n = 32, 26%), anxiety (n = 23, 18.7%), ADHD (n = 15, 12.2%), sleeping problems (n = 14, 11.4%), and autism spectrum disorder (n = 7, 5.7%).

### Psychometric analysis of the SIBS-T

Descriptive statistics of the six items of the *pre-version* of the SIBS-T tested in the pilot sample (n = 22) are displayed in Table 1. Means ranged from 2.16 to 3.03. For all items, all answer options (0–4) had been used. Athough according to the Kolmogorov Smirnov (K-S) test for all six items a normal distribution violation was found (*p* < 0.001), we assume an approximately normal distribution, since skewness for all items was < 2^21^. The corrected item-total correlations of all six items were acceptable^22^. However, item 4 (resistance) had a lower corrected item-total correlation (${r}_{it}$=0.46) than all other items (all ${r}_{it}$ > 0.50, Table 1). In addition, EFA showed an unacceptably low loading for item 4 (resistance). Moreover, internal consistency for the scale (Cronbach’s α) increased if item 4 was excluded (Table 1). Therefore, we decided to exclude item 4 (resistance) resulting in the final 5-item version of SIBS (for the final version of the scale, please consult supplementary material). To explore the relationship between SIBS-T and other characteristics, these five items (range, 0–4, each) were combined into a total SIB severity score (range, 0–20, the higher the score the more severe).

**Table 1:** Psychometric properties of the six items of the pre-version of the SIBS-T.

	Mean	SD	Item-total correlations	Factor loading	Cronbach’s α, if item excluded
1. Number	2.93	1.26	0.74	0.82	0.83
2. Frequency	3.03	1.45	0.77	0.86	0.83
3. Intensity	2.98	1.02	0.73	0.80	0.84
4. Resistance	2.75	1.27	0.46	0.48	0.88
5. Self-control	2.93	1.13	0.76	0.79	0.83
6. Impairment	2.16	1.14	0.58	0.62	86

Table 1 displays descriptive data, i.e., mean and SD for the six items of the *pre-version* of the SIBS-T, as well as corrected item-total correlations, factor loadings of an exploratory factor analysis and Cronbach’s alpha for the scale if the respective item was excluded. Item 4 was excluded from the questionnaire, so that only five items were included in the final version of the SIBS-T.

SIBS-T, self-injurious behavior scale for tic disorders; SD, standard deviation.

Psychometric properties of the 5-item scale were confirmed in n = 123 adults. Internal consistency of the SIBS-T was good (Cronbach’s α = 0.88 [0.85; 0.92]). In addition, a factor analysis confirmed a one-dimensional construct underlying the SIBS-T^23,24^. This one-factor solution explained 61% of the total item variance. Loadings of the items ranged from 0.63 (item 6) to 0.86 (item 2) with item 1 (0.80), item 3 (0.82), and item 5 (0.78), all showing good factor loadings.

To investigate convergent validity, only questionnaires assessing the same construct could be used but since there is no other instrument available measuring SIB in CTD, this could not be directly assessed. However, the SIBS-T showed medium correlations with the ATQ and the GTS-QoL, which would be expected for overlapping constructs. Irrespective of significance level, the SIBS-T showed only small correlations (< 0.3) with questionnaires assessing OCD, ADHD, anger attacks, depression, and anxiety, suggesting good discriminant validity with these questionnaires. The results for convergent and discriminant validity can be consulted in Supplementary Table 1.

### Type, intensity, and frequency of SIB

SIB was reported by n = 103 patients (83.7%) with a mean age of onset of 15.2 (SD = 8.46) years for SIB behavior and of 15.1 (SD = 9.05) years for SIB urge. Only two patients (1.9%) reported feeling an urge but never having performed SIB behavior and only n = 16 patients (13.0%) reported having ever performed a SIB without having felt an urge before.

On average, during the last four weeks, each patient suffered from 2.93 (SD = 1.26, range, 1–5) different SIB behaviors. The most frequently reported SIB behaviors (and also urges) were (in descending order) “beating/pushing/throwing”, “scratching”, “biting and licking”, and “tooth-related SIB” (Table 2). In contrast, the least frequent SIB behaviors (and also urges) were “trichotillomania” (TTM), “eye-related SIB”, and “self-burning” (Table 2).

**Table 2:** Frequencies of different categories of SIB urge and SIB behavior in patients with chronic tic disorders.

Type of SIB	SIB urge n/% (Mean; SD)	SIB behaviour n/% (Mean; SD)	Frequency in the whole sample (%)	
			Urge	Behaviour
Hitting/Pressing/Throwing			84	80
Hitting objects/wall with the head	56/46% (2.3; 0.81)	48/39% (1.8; 0.69)		
Hitting objects/wall with other body parts	70/57% (2.1; 0.88)	61/50% (1.6; 0.70)		
Hitting ones’ own head	67/55% (2.1; 0.89)	63/51% (1.5; 0.61)		
Hitting own body parts other than head	62/50% (2.2; 0.89)	60/49% (1.5; 0.56)		
Hitting head with objects	43/35% (2.5; 0.73)	34/28% (1.9; 0.74)		
Hitting other body parts with objects	40/ 33% (2.5; 0.75)	34/28% (1.7; 0.72)		
Hitting or pushing against different body parts with sharp objects	28 / 23% (2.7; 0.63)	13/11% (2.4; 0.78)		
Scratching and pinching			73	68
Pinching oneself	35/ 29% (2.6; 0.67)	31/25% (1.7; 0.72)		
Scratching oneself	44/ 36% (2.5; 0.77)	39/32% (1.7; 0.67)		
Scratching oneself that leads to bleeding	30/ 24% (2.7; 0.63)	26/21% (1.7; 0.73)		
Scratching open healing wounds	47/ 38% (2.4; 0.85)	45/37% (1.5; 0.58)		
Digging into ones’ skin with fingernails	42/ 34% (2.5; 0.75)	39/32% (1.7; 0.61)		
SIB related to eyes			35	30
Hitting own eyes	17/ 14% (2.8; 0.48)	12/10% (2.1; 0.72)		
Pressing against the eyes	26/ 21% (2.7; 0.58)	21/17% (1.9; 0.72)		
Hurting eyes with objects	3/2% (3.0; 0.27)	3/2% (2.3; 0.72)		
Biting and licking			72	68
Biting oneself	40/33% (2.5; 0.74)	35/28% (1.9; 0.62)		
Biting own lips	23/19% (2.8; 0.57)	19/15% (1.8; 0.76)		
Biting own tongue	14/11% (2.9; 0.44)	11/9% (2.0; 0.68)		
Cheek biting	22/18% (2.8; 0.58)	19/15% (1.6; 0.73)		
Fingernail biting	36/29% (2.5; 0.78)	36/29% (1.4; 0.50)		
Toenail biting	14/11% (2.8; 0.49)	11/9% (2.1; 0.62)		
Lip hurting due to constant lip licking	34/28% (2.6; 0.64)	34/28% (1.7; 0.49)		
Manipulation in the area of the teeth			55	46
Teeth grinding when awake	33/27% (2.6; 0.71)	27/ 22% (1.9; 0.71)		
Biting of objects	25/20% (2.8; 0.54)	13/11% (2.4; 0.58)		
Clenching teeth violently	37/30% (2.6; 0.71)	35/28% (1.7; 0.58)		
Manipulating teeth with hands	12/10% (2.9; 0.33)	8/7% (2.3; 0.62)		
Hurting teeth with objects	2/2% (3.0; 0.20)	2/2% (1.5; 0.71)		
Trichotillomania			21	20
Pulling out the hair from the head	14/11% (2.8; 0.53)	13/11% (1.6; 0.65)		
Pulling out eyelashes and/or eyebrows	8/7% (2.9; 0.36)	8/7% (1.6; 0.52)		
Pulling hair from other body parts	14/11% (2.8; 0.53)	13/11% (1.6; 0.65)		
Self-burning			37	30
Touching hot objects	37/30% (2.6; 0.69)	31/25% (1.9; 0.67)		
Self-burning	13/11% (2.9; 0.46)	9/7% (2.2; 0.60)		

SIB intensity during the last four weeks was rated by half of patients as mild (n = 51, 49.5%), by n = 28 patients (27.2%) as moderate, and only by n = 3 patients (2.9%) as severe, while 21 patients (20.4%) reported that they had either no SIB urge or an urge, but no SIB behavior during that time period.

With respect to SIB frequency during the last four weeks n = 28 patients (27.2%) reported that they experienced SIB several times a day, n = 7 (6.8%) once a day, n = 25 (24.3%) several times a week, n = 26 (25.2%) once a week or less, and a minority did not experience any SIB in this time period (n = 17, 16.5%) (frequency was not reported by all patients).

The second supplementary question (“How do you feel immediately afterwards when you give in to the urge and perform a SIB?”) showed that the majority of participants felt either somewhat better (n = 43, 41.7%) or neutral (n = 23, 18.7%) after SIB execution, while only a smaller number felt much better (n = 18, 14.6%). All other answer options were selected only by a minority: extremely better (n = 4, 3.3%), somewhat worse (n = 4, 3.3%), much worse (n = 5, 4.1%), and extremely worse (n = 2, 2%). Concerning the third supplementary question (“Do you feel a sense of pressure relief after SIB is performed?”) patients reported an average pressure relief of 62.8% (SD = 26.47) after SIB execution.

### Comparison of patients with and without SIB

When comparing patients with and without SIB, we found no differences with respect to demographic characteristics (age, sex) and the presence of pre-diagnosed psychiatric comorbidities (Table 3). However, severity of tics and severity of several psychiatric comorbidities including OCD, anxiety, depression, borderline personality traits and ADHD (according to self-assessments provided during data collection) were significantly higher in patients with SIB compared to those without. In addition, quality of life was significantly worse in patients with SIB (Table 3).

**Table 3:** Characteristics of patients with SIB (SIB+) compared to those without (SIB−).

Parameter		SIB+	SIB−	*t/chi*^*2*^	*p*	*d*
Demographics	Male, n (%)	67 (65%)	15 (75%)	0.86	0.651	
	Age M (SD)	35.95 (14.37)	39.5 (13.1)	− 1.03	0.308	− 0.25
Diagnosis	TS, n (%)	87 (84.5%)	15 (75%)	1.073	0.585	
	CMTD/CVTD, n (%)	10 (9.7%)	3 (15%)			
	UTD, n (%)	6 (5.8%)	2 (10%)			
Medication	Tic medication, n (%)	59 (57.3%)	10 (50%)	0.36	0.548	
Prediagnosed comorbidites	ADHD, n(%)	23 (22.3%)	3(15%)	0.54	0.463	
	OCD, n (%)	40 (38.8%)	5 (25%)	1.38	0.240	
	Depression, n (%)	59 (57.3%)	9 (45%)	1.02	0.312	
	Anxiety disorder, n (%)	31 (30.1%)	5 (25%)	0.21	0.647	
	ASD, n (%)	8 (7.8%)	3 (15%)	1.08	0.300	
	Sleep disorder, n (%)	21 (20.4%)	5 (25%)	0.21	0.644	
	Eating disorder, n (%)	12 (11.7%)	0 (0%)	2.58	0.108	
	Alcohol and/or substance abuse, n (%)	2 (1.9%)	1 (5%)	0.66	0.417	
	BPD, n (%)	5 (4.9%)	0 (0%)	1.01	0.314	
	Other personality disorder, n (%)	11 (10.7%)	1 (5%)	0.61	0.433	
Self-report questionnaires comorbidites	ATQ, M (SD)ATQ M (SD)	63.0 (36.0)	36.7 (18.9)	3.193.19	0.002*	0.78
	GTS-QoL, M (SD)	37.1 (21.0)	21.5 (17.9)	3.10	0.002*	0.76
	QoL-VAS, M (SD)	55.7 (20.8)	64.0 (23.0)	− 1.60	0.113	− 0.39
	OCI-R, M (SD)	18.3 (12.3)	6.6 (6.6)	4.13	< 0.001*	0.51
	ADHS-SB, M (SD)	19.5 (10.7)	10.7 (8.0)	3.50	< 0.001*	0.86
	BDI-II, M (SD)	14.4 (9.64)	10.0 (11.4)	1.82	.026*	0.45
	BAI, M (SD)	14.1 (10.6)	6.5 (8.6)	3.01	0.003*	0.74
	RAQ-R, M (SD)	18.9 (16.4)	12.3 (14.2)	1.61	.075	.39
	BSL-23, M (SD)	17.3 (16.6)	9.8 (11.9)	1.91	.01*	.47
	I-8, M (SD)	2.5 (1.19	2.0 (0.9)	1.95	0.53	.48

SIB, self-injurious behaviours; M, Mean; SD, standard deviation; TS, Tourette syndrome; CMTD, chronic motor tic disorder; CVTD, chronic vocal tic disorder; UTD, unspecified tic disorder; ADHD, attention deficit hyperactivity disorder; OCD, obsessive–compulsive disorder; ASD, autism spectrum disorder; BPD, borderline personality disorder; ATQ, adult tic questionnaire; GTS-QoL, Gilles de la Tourette Syndrome-Quality of Life Scale; QoL-VAS, quality of life visual analogue scale; OCI-R, obsessive–compulsive inventory-revised; ADHS-SB, ADHS-Selbstbeurteilungsbogen; BDI-II, Beck Depression Inventory-II; BAI, Beck Anxiety Inventory; RAQ-R, Rage Attack Questionnaire-Revised; BSL-23, Borderline Symptom List; I-8, Die Skala Impulsives-Verhalten-8.

Significant values after Bonferroni-correction are marked with an asterix, *d* – Cohens *d.*

### Correlations of SIB with tic severity and comorbidities

With respect to psychiatric comorbidities, we found strong Bonferroni-corrected correlations (significance-level: *p* = 0.008) between SIB as assessed by SIBS-T and tic severity, especially with the severity of vocal tics and tic complexity as per ATQ (Table 4). Similarly, we found positive Bonferroni-corrected (significance-level: *p* = 0.006) correlations between SIB according to SIBS-T and the severity of depression as measured with BDI-II and quality of life (according to GTS-QoL and QoL-VAS) (Table 5).

**Table 4:** Results of Pearson’s correlation between SIBS-T and ATQ.

	Total	Frequency	Intensity	Motor tics	Vocal tics	Simple tics	Complex tics
*r* (*p*)	0.34 (< 0.001)*	0.34 (< 0.001)*	0.32 (0.001)*	0.28 (0.004)*	0.38 (< 0.001)*	0.17 (0.086)	0.39 (0.001)*

ATQ, adult tic questionnaire, SIBS-T, self-injurious behavior scale for tic disorders.

Significant values after Bonferroni-correction are marked with an asterix.

**Table 5:** Results of Pearson’s correlation between SIBS-T and a variety of scales measuring severity of psychiatric comorbidities.

	RAQ-R	BAI	OCI-R	GTS-QoL	QoL-VAS	BDI-II	BSL-23	ADHS-SB
*r* (*p*)	0.08 (0.438)	0.14 (0.161)	.12 (.239)	0.40 (< 0.001)*	− 0.24 (0.015)	0.28 (0.005)*	0.24 (0.014)	0.26 (0.008)

GTS-QoL, Gilles de la Tourette Syndrome-Quality of Life Scale; QoL-VAS, quality of life visual analogue scale; OCI-R, obsessive–compulsive inventory-revised; ADHS-SB, ADHS-Selbstbeurteilungsbogen; BDI-II, beck depression inventory-II; BAI, beck anxiety inventory; RAQ-R, rage attack questionnaire-revised; BSL-23, borderline symptom list; SIBS-T, self-injurious behavior scale for tic disorders.

Significant values after Bonferroni-correction are marked with an asterix.

### Sex differences

In were unable to detect any differences between males and females with respect to the profile of comorbidities, demographic characteristics and SIB as assessed by SIBS-T.

## Discussion

We developed the first scale to assess severity of SIB in patients with TS/CTD. Factor analyses indicated good psychometric properties of the SIBS-T. Using the SIBS-T, we could demonstrate that SIB is a very frequent symptom in patients with CTD that affects nearly 85% of patients with no differences between males and females. SIB typically started in adolescence around the age of 15 years. SIB intensity during the last four weeks was rated by almost all patients as either absent or mild to moderate, and only by a minority as severe. While tic severity (measured with the self-assessment ATQ) was found to be strongly correlated with SIB severity, we failed to demonstrate any association between the presence of SIB and OCS/OCD. When analyzing the association between SIBS-T and ATQ subscores, we found that especially the subscore of complex tics was correlated with SIBS-T. Thus, based on our data, it is suggested that SIB represents a subtype of complex (motor) tics rather than a presentation of OCS. Patients with SIB had significantly higher scores of those scales measuring the severity of depression, ADHD, OCS/OCD, and anxiety indicating that patients with comorbid SIB suffer from more severe TS/CTD. In line with this, patients with SIB had significantly lower quality of life compared to those without.

Compared to recent studies, SIB prevalence found in our sample was much higher. While in previous reports, prevalence rates ranged between 4–43%^2–5,12,16^, in our sample of adults with TS/CTD, 85% of patients reported at least mild SIB. In a recent systematic review and meta-analysis by Stafford and Cavanna ^6^ the combined SIB prevalence from all studies published until 2020 was 35%. We believe that this discrepancy can be best explained as follows: (i) in recent studies, different definitions for SIB had been used. Therefore, it can be speculated that—depending on the definition used—only patients with more severe SIB had been included; (ii) this is the first study using a standardized measurement to assess SIB that included a detailed list of 32 different SIB behaviors. This procedure may have resulted in more extensive answers to the questions capturing also milder SIB. This interpretation is supported by the fact that half of patients in this study reported only mild severity of SIB and only very few patients severe SIB (defined as behaviors that led to permanent damage, injuries or scarring, or necessitated treatment by a physician); (iii) since SIB is defined as an “action to hurt oneself against one's own will” and has to be “carried out even though its senselessness and self-damage is recognized”, one may speculate that SIB is shameful and therefore patients suffering from SIB may tend to dissimilate this symptom in clinical interviews. Since this study was conducted online and completely anonymous, patients’ answers might have been more truthful; and (iv) while in most recent studies ^2–4^, mixed samples of children, adolescents, and adults had been included, in this study only adults (≥ 18 years) participated. In line with previous studies ^2^, mean age at onset of SIB was in adolescence. Thus, it can be assumed that prevalence rates in adults only samples are higher compared to mixed and children only studies.

In terms of phenomenology, similarly to previous reports^2,5,6,12,13,15,16^, the most frequent SIB found in our sample was self-hitting. Overall, the vast majority of patients reported mild to moderate SIB. During the last four weeks, on average patients reported three different SIB with a range from one to five.Based on our data, SIB should be classified as a subtype of complex tics instead of an OCS/OCD symptom or impulse control problem. Similar results were found in a study by Sambrani et al.^12^, but were not replicated in the other reports^2–6,12,18,25^ in which, along with an association with tics, SIB was also associated with OCD or impulsivity. These discrepancies could once more be the repercussion of different SIB definitions used and inclusion of diverse study samples^2–6,12,18,25^. Since complex tics occur more frequent in more severely affected patients suffering not only from more severe tics, but also from more comorbidities^12,16^, it is plausible that patients with comorbid SIB more often have psychiatric comorbidities including OCD, depression, anxiety, and ADHD. Furthermore, up to now it is unclear whether SIB indeed represents a consistent symptom. In particular, nail biting, lip licking, and TTM have alternatively been categorized under the umbrella of body-focused repetitive behavior (BFRB)^26^. In line with our data, these symptoms have also been described in tic disorders^27–29^. According to a recent large study from Taiwan including 535 children and adolescents with TS, 230 with provisional tic disorder (PTD), and 1,460 healthy controls^28^, in this age group of patients with TS the prevalence of nail biting was very high (56.6%) and significantly higher compared to PTD (27.4%) and controls (15%), respectively. Interestingly, nail biting started earlier than the tics. The authors of a recent review on TTM^29^ demonstrated that TTM is more related to tic disorders than to OCD. There is an ongoing discussion about the nature of BFRB that was previously classified as an impulse control disorder, later on as part of the OCD spectrum, while currently it has been suggested to be best classified as a manifestation of complex tics^29^. SIB categorization is further complicated by the fact that, similarly to tic disorders, SIB symptoms could belong to a spectrum ranging from very mild to severe cases^30^.

To the best of our knowledge, this is the first study investigating not only SIB behaviors, but also proceeding SIB urges. Similar to tics^31^, we found that 87% of patients report an urge before performing a SIB and nearly all patients some kind of pressure relief thereafter, further supporting the classification of SIB as a complex tic, rather than an OCS or impulse control problem. To differentiate whether this “pressure relief” is best explained as a relief similar to the momentary relief following the performance of a tic^31^ or should be interpreted as an emotional relief experienced following deliberate self-harm^32–34^ as seen in patients with BPD^35,36^, we additionally asked how patients “feel” immediately after having performed SIB. On average, patients indicated feeling only “minimally improved” after having performed SIB, which is in contrast to reports of patients with deliberate self-harm, where “feeling better” is one of most commonly reported motives for this behavior^32^.

Our study has several limitations. Firstly, the study was conducted online, and all variables were self-reported and, therefore, are not entirely reliable and were not confirmed by medical professionals. We also could not confirm inclusion and exclusion criteria. Also, only patients with internet access were included. It cannot be entirely excluded that patients participated more than once and/or provided untruthful information. Furthermore, a selection bias cannot be excluded, since participants were aware of the primary purpose of the study and therefore it is possible that mainly patients with comorbid SIB participated. Secondly, this study was based on data from a single center in Germany and it cannot be excluded that findings may differ depending on the cultural context. Thirdly, since SIB may occur not only in patients with TS/CTD, but also in psychiatric disorders such as BPD, we re-analyzed data after exclusion of those five patients who stated that they had been pre-diagnosed with BPD. In addition, we re-analyzed data after item 5 of the BSL-23 (“During the last week I thought about self-injury”) was eliminated to prevent unintentional confounding with SIB. This procedure yielded the same pattern of results (data not shown) so that we do not believe that results are biased due to comorbid BPD. Fourthly, although the study sample was relatively large, our findings should be replicated in an independent larger study group. Fifthly, it cannot entirely be excluded that our sample is biased by patients suffering form functional tic-like behavior (FTLB) or coexisting functional symptoms, which is a common comorbidity in TS^37^ , since data were collected via an online survey. Because self-hitting is a well-known symptom in these patients^38^, we only asked people to participant, in case the diagnosis of TS/CTD had been confirmed by a physician or psychologist. In addition, we asked participants by whom the diagnosis had been made. We believe that influence on data by FTLB is minimal, since the majority of patients was recruited via our clinic, 60% of patients reported having been diagnosed at our or another German TS outpatient clinic, and altogether 94% of patients indicated that the diagnosis had been made by a neurologist, psychiatrist, child and adolescent psychiatrist, or psychologist. Sixthly, although overall SIB was found to correspond to the spectrum of tics, it cannot be excluded that at least some behaviors might be better classified under the plethora of OCD, particularly as it is unknown, whether SIB in TS represents a consistent diagnostic category. In order to determine this, a detailed network or cluster analysis could be done using larger sample sizes. Seventhly, we have not investigated test–retest reliability. To further assess psychometric quality of the SIBS-T, in future studies consistency and reproducibility of results should be investigated. Eighthly, we did not include patients in the planning of the study and the very first step of the scale development. However, we aimed to incorporate active patient engagement in the further development of the SIBS-T and therefore performed two pre-tests asking for missing SIB and supplementary information related to SIB, which provided useful data for the development of the final version of the SIBS-T.

## Conclusions

In summary, in this paper we present the first scale to assess SIB in patients with tics. We were able to demonstrate good psychometric properties of the SIBS-T. Using the SIBS-T, we showed that SIB is more frequent in patients with TS/CTD than previously reported further supporting future use of this validated instrument. SIB significantly impairs patients’ quality of life. Based on our data, SIB constitute a type of complex tic rather than an OCS or impulse control problem. Accordingly, anti-tic medications should be a more appropriate approach to treat SIB than treatment with selective serotonin reuptake inhibitors.

## Methods

### Definition of SIB

For the purpose of this study, we decided to apply the term “self-injurious behavior” (SIB) as this is the most widely used nomenclature with respect to self-harming behavior in patients with TS. In addition, this term follows the diagnosis listed in the 5th edition of the Diagnostic and Statistical Manual of Mental Disorders^39^, where self-injury is differentiated in suicidal and non-suicidal behavior^40^. According to the International Society for the Study of Self-Injury, SIB is defined as deliberate, non-suicidal behavior, which is not socially sanctioned^41^. Following existing definitions given in the literature^2–4,6,40–42^ and based on clinical experience, we established the following definition of SIB: “Self-injurious behavior (SIB) is defined as an urge to or a behavior of injuring oneself against one’s own will. SIB are not performed intentionally and are not the result of an accident. They can lead to injury or harming of one’s own body without having an intension of self-harm or suicide (suicidality). These behaviors must be performed even though their senselessness and self-damage are recognized”.

It is worth noting that in the German version of the survey and the SIBS, we decided to use the German term “Autoaggression” (in English: autoaggression), since in German language the term “self-injurious behavior” (in German: selbstverletzendes Verhalten) is primarily used and established in the context of Borderline personality disorder (BPD). By using the term autoaggression, we wanted to avoid a bias due to this close association (for further precautions to avoid influences by comorbid disorders see below).

### Structure of the survey, item development, and pre-test

Based on this definition, the SIBS-T was developed by an interdisciplinary team (neurologists, psychiatrists, and psychologists), some of whom (KMV, EJ) have several years of clinical and research experience in TS. The SIBS-T provided detailed information about different types of SIB allowing further classification into subgroups and identification of main symptom clusters. Structurally, the SIBS-T was based on the Yale Brown Obsessive Compulsive Scale (Y-BOCS)^43^, the gold standard for the assessment of obsessions and compulsions in OCD, since (i) the Y-BOCS is a reliable and validated measurement and (ii) parallels between SIB and OCD have been demonstrated in previous studies^2,3,5^.

After consultation by four external TS experts from Germany and the UK and appropriate adjustments, a preliminary version of the SIBS-T was sent to 10 patients with TS to determine whether the questions are clear enough and asking for feedback. After appropriate item adjustment, this pre-version was piloted online from 07.04.2020 to 30.04.2020 in 22 participants with TS using the online platform SoSciSurvey^46^ in order to further involve patients in the scale development by evaluating the scale and providing feedback to nine additional questions in the pre-version of the SIBS-T.

The pre-version of the SIBS-T consisted of three parts: (i) a symptom checklist including – similarly to the Y-BOCS—32 most frequently described SIB^2,3,6,11,12,20,44,45^ (see Table 2 for individual SIB) clustered in the following seven categories: hitting/pushing, scratching/pinching, eye-related SIB, biting/licking, tooth-related SIB, TTM, and skin burning. Since so far no clear definition of SIB was available, we included in the symptom checklist those SIB behaviors that were most commonly reported in TS in the literature. In addition, however, we asked participants to add any SIB behavior that they felt was missing. For each of the 32 SIB, participants were asked to indicate whether they experience the symptom currently (during the last four weeks) and/or in the past; (ii) six items assessing different aspects of SIB including number (item 1), frequency (item 2), intensity (item 3), resistance (item 4), control over SIB (item 5), and impairment (item 6), each rated on a scale ranging from 0 to 4; and (iii) nine supplementary questions, to better understand the characteristics and to further improve the definition of SIB (age of onset of SIB urge and SIB behavior, feelings related to SIB behavior (improved, worse, relaxed, satisfied, or relieved), occurrence of SIB behavior without a preceding urge, awareness of senselessness, risk of injury of SIB, and fear of SIB occurrence or related injuries. Thus, the total score of the *pre-version* of the SIBS was calculated by summing up the six items assessing different SIB aspects (range, 0–24).

Since we were interested in both SIB behavior and preceding urges, participants were asked to complete the 32-item symptom checklist three times: firstly, indicating for which of the 32 SIB an urge was felt ever (independently of a following SIB behavior); secondly, for those SIB, for which it was stated that an urge had ever been felt—either currently or in the past—it was asked, whether this SIB had actually ever been performed; and thirdly, indicating whether any of the SIB had ever been performed *without* a preceding urge.

The results of the pilot data confirmed the aforementioned definition of SIB, since the majority of patients (82%) indicated feeling an urge prior to SIB behavior and only 27% stated that they had performed a SIB without feeling a preceding urge. Almost all patients (91%) were aware of the senselessness of their SIB and a substantial number (86%) of its possible harm. Remarkably, nearly 2/3 of patients (63%) were afraid of hurting themselves by a SIB. However, based on these results it remained unclear, whether performing a SIB makes oneself feel better (50% answered “yes”) or worse (28% answered “yes”) or results in a pressure relief (55% answered “yes”). In the pre-version of the SIBS-T, we decided for a time period of two weeks to be assessed (in contrast to the Y-BOCS using a period of one week), since we assumed that SIB may occur less commonly than tics and OCS. However, 36% (8/22) of patients indicated that they did not have any SIB behavior during the last two weeks. Therefore, we decided in the SIBS-T for a longer time period of four weeks. According to participants’ suggestions, we added three further SIB behaviors in slightly modified form in the final version of the SIBS-T: “jab sharp objects against or into my body”, “press my fingernails hard into my skin”, and “bite hard into/on hard objects so that my teeth could have sustained damage” (for the final version of the scale, please consult supplementary material).

After the evaluation and interpretation of the results of the pilot questionnaire, we decided to add three supplementary questions with revised wording to the final version of the survey to further investigate different aspects of SIB: (i) age of onset of SIB urge and SIB behavior; (ii) “How do you feel immediately afterwards when you give in to the urge and perform a SIB?” (rated on a seven-point scale: 1 = very much improved; 2 = much improved; 3 = minimally improved; 4 = no change; 5 = minimally worse; 6 = much worse; 7 = very much worse), and (iii) “Do you feel a sense of pressure relief after SIB is performed?” (0–100%, 0% = no pressure relief at all, 100% = complete pressure relief).

### Study design and assessments

This study was a prospective, cross-sectional study conducted exclusively online via SoSciSurvey. All data were collected anonymously. All aspects of data protection were guaranteed. We collected demographic data (sex, age, level of education), asked for current medication and other treatments, and collected information about previously diagnosed psychiatric disorders, i.e., ADHD, OCD, OCS, depression, anxiety, sleep disorder, eating disorder, alcohol or drug addiction, and personality disorders, especially borderline personality disorder. If any psychiatric comorbidity had been diagnosed, participants were asked whether symptoms are still present and clinically relevant.

In addition, a number of self-administered assessments were used to measure severity of tics and comorbid symptoms: (i) Adult Tic Questionnaire (ATQ) for assessment of tics^47^, (ii) Self-Assessment Scale of ADHD (ADHS-Selbstbeurteilungsbogen, ADHS-SB) for assessment of ADHD^48^, (iii) Obsessive–Compulsive Inventory-Revised (OCI-R)^49^ for evaluation of OCS/OCD, (iv) Scale of Impulsive Behaviors (Skala Impulsives-Verhalten-8, I-8)^50^ for assessment of impulsiveness, (v) Beck Depression Inventory (BDI-II)^51^ for evaluation of depression, (vi) Beck Anxiety Inventory (BAI)^52^ for evaluation of anxiety, (vii) short version of the Borderline Symptom List (BSL-23)^53^ for assessment of features typical for borderline personality disorder, and (viii) Rage Attack Questionnaire-Revised (RAQ-R)^54^ for evaluation of rage attacks. For evaluation of quality of life, Gilles de la Tourette Syndrome Quality of Life Scale (GTS-QoL)^55^ and Visual Analogue Scale (QoL-VAS)^56^ were used.

### Inclusion criteria

The following inclusion criteria were defined: (i) diagnosis of a chronic (> 1 year) tic disorder (TS, CTD, or unspecified tic disorder) confirmed by a physician or psychologist self-report, (ii) age ≥ 18 years, (iii) fluent in German language, and (iv) internet access. To verify as much as possible that only patients with pre-diagnosed chronic tic disorders participated in this study, in addition, we asked for age of diagnosis, type of tic disorder, and the diagnosing expert (e.g. neurologist, psychiatrist, family doctor). Patients with and without known SIB were asked to participate.

### Recruitment

After approval from the local ethics committee (no. 9010_BO_K_2020), patients were recruited via the Tourette Outpatient Clinic at the Hannover Medical School (MHH) and German self-help groups (Tourette Gesellschaft Deutschland e.V., Interessenverband Tic & Tourette-Syndrom, Austrian Tourette Society, Swiss Tourette Society, „LifeTiccer”, and the Tourette Online site https://www.tourette.de). Data were collected between June and October 2020.

### Statistical analysis

#### Sample calculation

According to the sample size calculation, n = 40 subjects are required to detect an effect if it was there (assumed sample mean > 0.65), using a t-test with a two-sided significance level of 5% with a power of 80%. We used a non-probability sampling approach, i.e. all patients that agreed to participate from 08.06.2020 until 31.10.2020 were included in the study.

### Psychometric analysis of the SIBS-T

Statistical analyses were performed using SPSS 20.0 software. With regard to item selection, corrected item-total correlations were calculated with the minimum of 0.3 recommended by Lienert and Raatz^57^. An Exploratory Factor Analysis (EFA)^58^ was conducted to assess the unidimensional nature of the scale, using the syntax written by O’Connor^24^. In order to assess the internal consistency of the scale, an indicator of the reliability of a questionnaire, Cronbach’s α, was calculated. A value of α > 0.80 is generally considered as good. In addition, scale reliability was assessed with each item extracted to determine whether an item should be removed^59^. To test for convergent and discriminant validity, we have conducted a Pearson’s correlation between SIBS-T and other measurements used in the study (for further details see Supplementary Table 1)^60^.

### Comparison of patients with chronic tic disorders with and without SIB

Group comparisons of SIB + (with) and SIB- (without) were performed considering a number of demographic and clinical variables including age, sex, medication, and prediagnosed psychiatric comorbidities. The Shapiro–Wilk test was used to assess the normality of the item distribution. Chi-square and Fisher tests were performed for comparison of categorical variables. The Mann–Whitney U test was used to contrast numeric variables. T-tests were used to assess self-reported diagnoses of comorbidities. The *p*-value was Bonferroni-corrected (0.05/13 questionnaires) and only considered significant if *p* < 0.004. Pearson’s correlations were used to evaluate the associations between SIBS-T and clinical variables in the whole sample.

To examine the relationship between SIB and tic severity, comorbidities, and quality of life, Pearson's correlations were performed. For more detailed investigations regarding the relationship between tic severity and SIB, the ATQ was categorized into motor/vocal tics, simple/complex tics, complex motor/complex vocal tics, by frequency and intensity, and by mild/moderate/severe severity levels, and Pearson's correlations were performed again. All tests were Bonferroni-corrected (significance level divided by the number of tests conducted).

### Ethical approval

All methods were carried out in accordance with relevant guidelines and regulations.Informed consent was obtained from all subjects.

### Supplementary Information

Supplementary Information 1.

Supplementary Information 2.

## Data Availability

The datasets used and/or analysed during the current study available from the corresponding author on reasonable request.
