# Supplementary material for: Validation and assessment of the self-injurious behavior scale for tic disorders (SIBS-T)
Source: Sci Rep. 2024 Jul 31;14:17727. doi: 10.1038/s41598-024-66528-6 (PMC11291896; doi:10.1038/s41598-024-66528-6)
Supplement: Supplementary file 1 — Supplementary Information 1. [file 41598_2024_66528_MOESM1_ESM.docx]

Supplementary material.

**Self-Injurious Behavior Scale for Tic Disorders (SIBS-T)**

# Definition

"Self-injurious behavior (SIB) is defined as an urge to or a behavior of injuring oneself against one’s own will. SIB are not performed intentionally and are not the result of an accident. They can lead to injury or harming of one’s own body without having an intension of self-harm or suicide (suicidality). These behaviors must be performed even though their senselessness and self-damage are recognized”.

# Instructions

Various SIB are listed below that occur more frequently in people with Tourette syndrome and other chronic tic disorders. Please indicate for each of the SIB listed below whether you have actually PERFORMED these “CURRENTLY” (i.e. in the past 4 weeks) and/or “IN THE PAST”. If you have performed it both in the past and currently, please put both (“Currently” and “In the past”).

**I have previously, against my will, had to…**

... hit my head against objects/wall

... hit other body parts against objects/wall

... hit my own head

... hit myself against other body parts (such as arms)

... hit my head with objects or press them against my head

... hit my eye

... press on, into or behind my eye

... injure my eye with objects

... hit other body parts with objects or press objects against them

... jab sharp objects against or into my body

... pinch myself

... scratch myself

... bite myself

... scratch my skin until it bleeds

... keep scratching wounds open

... press my fingernails hard into my skin

... injure my lips by constantly licking them (“cracked lips”)

... bite my lips so that they could have sustained

wounds/injuries

... bite my tongue so that it could have sustained wounds/injuries

... bite my cheeks so that they could have sustained wounds/injuries

... pull out the hair on my head

... pull out my eyelashes or eyebrows

... pull out hair elsewhere on my body

... bite hard into/on hard objects so that

my teeth could have sustained damage

... rub my teeth together when I am awake so that my teeth could have sustained damage

... hit my teeth together hard

... loosen my teeth with my hands

... damage my teeth by hitting them with objects or pressing objects against them

... touch hot objects (such as an oven, a stove, candle flame, cigarette)

... inflict burns on my skin

... bite my fingernails or manipulate them so that they could have sustained injuries

... bite my toenails or manipulate them so that they could have

sustained injuries

# Assessment of SIB

Please assess your SIB by selecting the statement that best reflects your situation.

Note: To answer the following questions, please take into account any SIB that have occurred during the past 4 weeks.

1. **How many different SIB have you performed in the past 4 weeks?**
2. 0
3. 1
4. 2-3
5. 4-5
6. >5
7. **How often have SIB occurred in the past 4 weeks?**
8. Not at all
9. Once a week or more seldomly
10. Several times a week
11. About once a day
12. Several times a day

1. **How pronounced have your SIB been in the past 4 weeks?**
2. There were neither SIB behaviours nor an urge to perform such behaviours
3. There was an urge to perform SIB without me having to actually perform them
4. There were mild SIB without consequences that were visible or that were noticeable to others
5. There were moderate SIB that led to superficial or only minor injuries but did not necessitate further medical treatment
6. There were severe SIB that led to permanent damage, injuries or scarring or necessitated treatment by a doctor
7. **How severely have you been impaired in the past 4 weeks by your SIB?**
8. There was no impairment.
9. There were minimal difficulties in relation to self-esteem, family life, social acceptance, performance at school/in the workplace or health. For example, occasional worries about the future and my own health, slight family tensions or fear that others could notice the behavior.
10. There were slight difficulties in relation to self-esteem, family life, social acceptance, performance at school/in the workplace or health. For example, feelings of guilt and shame, occasional pain, a guilty conscience, or reproaches on the part of family/friends.
11. There were clear problems in relation to self-esteem, family life, social acceptance, performance at school/in the workplace or health. For example, mood swings, conflicts in the family, visible scarring/bruising/wounds, teasing and marginalization, loneliness due to the avoidance of social contact or deteriorating performance.
12. There were extremely severe difficulties in relation to self-esteem, family life, social acceptance, performance at school/in the workplace or health. For example, depression, self-hatred, family breakdown, job loss, school dropout, social isolation or permanent physical damage.
13. **How much control have you had in the past 4 weeks over your SIB? How successful have you been thereby in ending or suppressing your SIB?**
14. I had full control over my SIB
15. I had strong control over my SIB and could usually end or suppress them with some effort and concentration
16. I had moderate control over my SIB and could sometimes end or suppress them
17. I had little control over my SIB and could seldom successfully end or suppress them
18. I had no control over my SIB and could neither successfully end them nor fully suppress them

**TOTAL SIBS-T SCORE (sum of the individual scores): 0-20**
