# Supplementary material for: Validation and assessment of the self-injurious behavior scale for tic disorders (SIBS-T)
Source: Sci Rep. 2024 Jul 31;14:17727. doi: 10.1038/s41598-024-66528-6 (PMC11291896; doi:10.1038/s41598-024-66528-6)
Supplement: Supplementary file 2 — Supplementary Information 2. [file 41598_2024_66528_MOESM2_ESM.docx]

**Supplementary Table 1: Estimation of convergent and discriminative validity of SIBS-T.**

| **Convergent validity** | **Divergent validity** | **Pearson correlation (r)** | **p** |
| --- | --- | --- | --- |
| RAQ-R  OCI-R  ATQ  GTS-QoL  QoL-VAS  BDI-II  ADHS-SB  I-8  -Urgency  -Lack of premeditation  -Lack of perseverance  -Sensation seeking | BAI  BSL-23 | 0.098  0.134  **0.305**  **0.383**  **-0.244**  **0.264**  **0.264**  -0.063  -0.081  **-0.263**  -0.097    0.136  **0.225** | 0.325  0.177  **0.002**  **< 0.001**  **0.013**  **0.007**  **0.007**  0.525  0.414  **0.007**  0.329  0.172  **0.022** |

SIBS-T – Self-Injurious Behavior Scale for Tic Disorders, RAQ-R - Rage Attack Questionnaire-Revised, OCI-R - Obsessive-Compulsive Inventory-Revised, ATQ – Adult Tic Questionnaire, GTS-QoL - Gilles de la Tourette Syndrome-Quality of Life Scale, QoL-VAS - Quality of Life Visual Analogue Scale, BDI-II - Beck Depression Inventory-II, ADHS-SB - ADHS-Selbstbeurteilungsbogen, I-8 - Skala Impulsives-Verhalten
